# Musculoskeletal disorders in video gamers – a systematic review

**DOI:** 10.1186/s12891-022-05614-0

**Published:** 2022-07-16

**Authors:** Chuck Tholl, Peter Bickmann, Konstantin Wechsler, Ingo Froböse, Christopher Grieben

**Affiliations:** 1 Institute of Movement Therapy and Movement-oriented Prevention and Rehabilitation, German Sport University Cologne, Cologne, Germany; 2 Institute for Occupational Safety and Health of the German Social Accident Insurance, Sankt Augustin, Germany; 3 University of Applied Management, Ismaning, Germany

**Keywords:** Video gaming, MSD, Sedentary behavior, Physical pain, Esports

## Abstract

**Background:**

Video gaming is a recreational activity with yearly increasing popularity. It is mostly a sedentary behavior combined with repetitive movements of the upper limbs. If performed excessively, these movements may promote strain injuries and a sedentary lifestyle is one of the contributing factors to musculoskeletal disorders. Therefore, a systematic review was conducted to evaluate if video gaming negatively affects the musculoskeletal system of video gamers.

**Methods:**

PubMed, Web of Science and The Cochrane Library were systematically searched in order to identify relevant peer reviewed original articles in English published between 2000 and 2021. The Preferred Reporting Items for Systematic Reviews and Meta-Analyses (PRISMA) method was used for the analysis. Studies were included when they contained investigations of changes of the musculoskeletal system due to video gaming in healthy individuals. Studies with participants older than 60 years or solely psychological, social or cardiovascular outcomes were excluded. An adapted version of the Newcastle–Ottawa Scale was used for the risk of bias analysis.

**Results:**

Sixteen observational studies involving a total of 62,987 participants met the inclusion criteria. A majority (11) of the studies reported statistical negative musculoskeletal changes due to video game playtime. Four studies did not report changes and one study found no effect of video game playtime on the musculoskeletal system. Out of the eleven studies, which demonstrated a negative impact of video game playtime on the musculoskeletal system, the most reported painful body parts were the neck (*n* = 4), shoulder (*n* = 4) and back (*n* = 3). Ten studies reported odds ratios (OR) for the dependence of the appearance of musculoskeletal disorders on video game playtime. In eight studies OR were significantly increased (1.3—5.2).

**Conclusion:**

Eleven out of twelve studies demonstrated a negative impact of video game playtime on the musculoskeletal system. In particular, excessive video game playtimes (> 3 h/day) seemed to be a predictor for the appearance of musculoskeletal disorders. Due to their great popularity across multiple generations, specific and tailored prevention and health promotion programs for video gamers need to be developed to counteract this important public health issue.

**Supplementary Information:**

The online version contains supplementary material available at 10.1186/s12891-022-05614-0.

## Introduction

Video gaming is one of the most popular recreational activities in the world [1]. In 2021 there were approximately 3.24 billion video gamers across the globe [2]. Alone Asia had 1.48 billion video gamers followed by Europe with a gaming audience of 715 million [2]. For the past several years the global video gaming industry has been growing. This has resulted in a global market amount close to USD 176 billion in 2021 [3]. This is almost twice as much as the market size of the global fitness and health club industry in 2019 [4], which is also a big part of the leisure activity sector. Therefore, video gaming cannot be seen as a short-term trend. However, it is important to keep in mind that especially the video gaming industry was one winner of the worldwide corona pandemic [5], because video gaming was not restricted by it. On the contrary, a lot of people had more leisure time and they spent their free time on video gaming in the corona lockdowns around the globe [5, 6], which was a perfect basis for an accumulation of sedentary time.

Video gaming is a screen-based activity, comparable to watching TV, working at the computer or using a smartphone. These kinds of activities are characterized by long, continuous sitting periods and physical inactivity [7]. Such sedentary behaviors are well-known risks of all-cause mortality and non-communicable diseases (NCD) [8, 9]. Watching TV is probably the most motionless undertaking of the screen-based activities, because it typically occurs in the evening time after dinner and a day of work [10]. Therefore, it is a kind of relaxing activity for most people [11]. Working at a computer, e.g., in the office on the other hand, often involves different kinds of sitting or standing positions, which are occasionally interrupted with short walks [12]. In addition to this seated position, video gaming requires fine motor skills to execute the gaming process [13, 14]. Therefore, it involves repetitive movement of the arms, wrists, hands and fingers in order to interact in the virtual environment [15], similar to some types of office work or even more intense activities [16]. Such movements, when performed continuously and without interruption, can lead to musculoskeletal disorders (MSDs), especially of the upper limbs [17, 18]. Moreover, MSDs are associated with physical inactivity, prolonged sitting and unilateral (sitting-) postures [19].

MSDs are a huge financial burden for healthcare systems in European countries. In 2016 Germany reported a loss of gross value added of EUR 30.4 billion because of musculoskeletal and connective tissue disorders [20]. This represents 1.0% of Germany’s gross domestic product (GDP). The Swedish public health care reported approximately 20–30% of all visits were caused by MSDs and in France annual costs borne by enterprises exceed EUR 1 billion per year through MSDs [20]. Thus, in order to reduce health care costs and increase productivity, all countries should be interested in reducing the prevalence of MSDs. The generic term musculoskeletal disease includes several different kinds of conditions and diseases which affect bones, joints, muscles, and connective tissues [21]. For this reason, there is not only one but a variety of definitions of MSDs. Due to this diversity of definitions and thus a wide range of included diseases, the evaluation of MSDs lacks in a gold standard approach. Therefore, many different evaluation methods have been used. The most common ones are subjective (non-)standardized questionnaires, which are used to evaluate pain or discomforts in a defined time period [22]. Alternatively, (objective) clinical tests such the Finkelstein’s test [23], have been used to evaluate single musculoskeletal issues. Thus, a wide variety of diseases and injuries have been listed and evaluated under the term MSD. The main physical causes for MSDs are lifestyle factors, being overweight, physical (non-)activity, postural problems, sports, heavy workload and ergonomic aspects [20, 24]. In addition, psychosocial, socioeconomic and environmental risk factors can be important [20]. Video gaming combines many of these factors like prolonged sitting-periods with a lack in physical activity, repetitive movements of the upper limbs and ergonomic burdens. Consequently, video gaming can be expected to lead to a higher risk for MSDs. The results of previous research have indicated this association [15, 25, 26]. Nevertheless, at this moment there is no overall-evidence of whether video gaming could affect the musculoskeletal system negatively.

The aim of this systematic review is to give an overview and review evidence of the extent to which video gaming influences the musculoskeletal system of gamers. For this purpose, in this systematic review it was investigated (1) whether video gaming negatively affects the musculoskeletal system of gamers, (2) to what extent the factor playtime influences musculoskeletal changes and (3) if there are certain body parts or specific tissues particularly affected. This is especially important if the imbalances in video gamer’s health-behavior and special needs are to be identified. Moreover, these findings could be used for group specific prevention and rehabilitation recommendations to counteract these behaviors and improve the health of video gamers.

## Methods

The protocol for this review was registered and published in the PROSPERO database (CRD42021220167) and was reported in accordance with the Preferred Reporting Items for Reviews and Meta-Analysis (PRISMA) [27].

### Eligibility criteria

Studies had to be peer-reviewed, published between 2000 and 2021, and written in English. Participants in the studies had to be active video gamers.

All studies in which changes were investigated in the musculoskeletal system because of video gaming were of interest. This included issues of bones, cartilages, ligaments, tendons, muscles and tissues. Of special interest were negative changes in all human tissues and movements, for example range of motion, muscle tensions, pain syndromes and injuries. Reports about changes in the posture, muscle-nerve-connectivity or pain were included, as well as studies of the assessment of harms like cross-sectional, case–control or longitudinal studies. In addition, experimental studies like randomized controlled trials (RCTs) were also included.

Studies were excluded if they only contained psychological, social, or cardiovascular outcomes. Other reviews, meta-analysis and case reports were excluded for this review. Moreover, studies on overweight or obese people were excluded. Studies with elderly people (over 60 years) were excluded, along with studies which were restricted to specific musculoskeletal diseases or conditions or psychological, cognitive or social disorders.

### Information sources and search strategy

Systematic electronic literature searches were undertaken of PubMed, Web of Science and Cochrane Library databases. Additionally, Google Scholar and reference lists of included studies were searched unsystematically. The first systematic electronic literature search was conducted on 08 October 2020 using PubMed and was updated on 01 December 2020 and 26 June 2021. For Web of Science the first search was conducted on 20 October 2020 and was updated on 02 December 2020 and 26 June 2021. The Cochrane Library was searched on 15 December 2020 and this was updated on 26 June 2021. Unsystematic searches of Google Scholar were conducted on 08 and 09 December 2020. Literature search strategies used medical subject headings (MeSH) and text words related to video gaming and musculoskeletal, injury or pain (Additional file 1).

### Selection process

Author 1 and 2 independently screened titles and abstracts to include studies for full text analysis. The full text of the included studies were also independently reviewed by both authors to decide on inclusion or exclusion. Author 3 helped to find a consensus solution for the cases where there was a disagreement.

### Data collection process

The systematic electronic literature search results were uploaded to Citavi 6 [28], a reference management program, to determine if there were duplicates. After that, literature was uploaded to Rayyan [29], an internet-based software which facilitates collaboration among reviewers during the study selection process. As the final step, a manually created excel sheet was used for data extraction from the included studies.

### Study risk of bias assessment

An adapted version of the Newcastle–Ottawa Scale (NOS) for cross sectional studies [30, 31] was used to evaluate the risk of bias for the included studies. The scale included four items for selection (maximum 5 stars), one item for comparability (maximum 2 stars) and two items for outcome (maximum 3 stars). Therefore, each selected study was rated with a number from 0 (low quality) to 10 stars (high quality). By definition, a total of 7 or more stars indicated high quality, 4 to 6 stars indicated medium quality and 3 or less stars indicated low quality. Author 1 and 2 independently rated the studies and differences were resolved after a discussion. If there were further disagreements regarding study ratings Author 3 was involved in order to find a consensus solution.

### Synthesis of results

It was anticipated that the identified studies in this systematic review would be heterogeneous in the study design. Therefore, the study results were synthesized narrative without any further statistical analysis. The narrative synthesis is based on the guidelines of the Centre for Reviews and Dissemination [32].

## Results

### Study selection

**Fig. 1:**
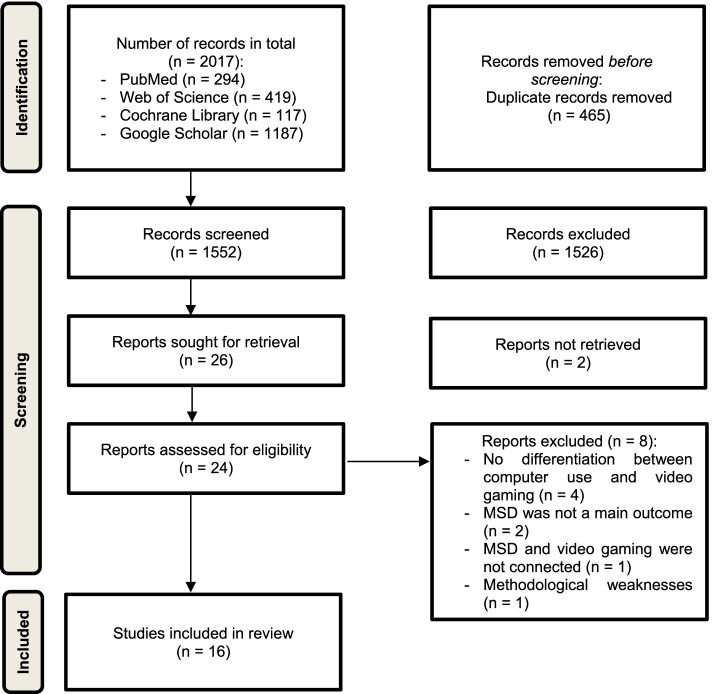
PRISMA Flow chart of study selection

In Fig. 1: PRISMA Flow chart of study selection it is shown, that a total of 2017 articles were identified in three databases and Google Scholar. After removing duplicates (465), the titles and abstracts of the remaining 1552 studies were screened, of these 1526 studies did not fit with the inclusion criteria. Thus, a total of 26 full text articles were assessed for their eligibility. Finally, 16 eligible studies were included for the analysis.

### Study characteristics

In Table 1: Study characteristics the characteristics are shown of the 16 included studies. A total of 62,987 participants from ten different countries and four continents were involved. The dominant population in eleven out of 16 included studies, were school aged kids and young adults aged between 5 to 20 years. Studies were mainly cross sectional, with the exception of one experimental study [26]. Out of the four possible video gaming devices (computers, consoles, handhelds, smartphones), seven studies included one device [25, 26, 33–37], three studies included two devices [38–40] and six studies included three or more devices [41–46]. Due to insufficient information, it is not possible to rule out that in two studies [37, 39] more than the indicated devices were used. Overall computers were the most commonly reported devices in 13 studies. The main body regions of reported musculoskeletal pain were the neck (*n* = 7), back (*n* = 6), shoulders (*n* = 5) and hands (*n* = 5). As the evaluation method, in eleven of the studies self-designed non-standardized questionnaires were used [25, 36–39, 41–46]. Moreover, in two studies standardized questionnaires or checklists were used [33, 40] and one study each involved the use of electromyography (EMG) data and a visual analog scale [26], a clinical test and a partially standardized questionnaire [34] or only a partially standardized questionnaire [35].

**Table 1:** Study characteristics

First author (year), country	Study design	Population	Sample size (n)	Mean Age [yrs] (range), sex	Types of video gaming device^a^	Definition of MSD to identify changes^b^	Evaluation methods	Body regions of interest
**Burke (2002), USA **[41]	Cross sectional survey	School students	212	12.4 (5 – 18), 52% female	Computer, console, handheld	Presence of cumulative trauma disorders	Non-standardized questionnaire	Back, head, neck, wrist
**DiFrancisco-Donoghue (2019), USA **[37]	Cross sectional survey	College esport players	65	NR (18 – 22), NR	Computer, more CD	Reported pain in different body regions	Non-standardized questionnaire	Back, hand, neck, wrist
**Hakala (2006), Finland **[38]	Cross sectional survey	School students	6003	14-, 16-, 18-year-olds, 52.2% female	Computer, console	Neck-shoulder pain [NSP] or lower back pain [LBP] in the past 6 months	Non-standardized questionnaire	Back, neck, shoulder
**Hellström (2015), Sweden **[39]	Cross sectional survey	School students	7757	13–14-, 15–16-, 17–18-year-olds, 50% female	Computer, console, more CD	MSK pain during the past 3 months	Non-standardized questionnaire	Summarized^3^
**Kang (2003), Korea **[33]	Cross sectional survey	Gaming room visitors and college students	284	22.9 (17—29), 100% male	Computer	Presence, duration and severity of MSDs in upper limbs	NIOSH Criteria	Elbow, hand, neck, shoulder, wrist
**Lindberg (2020), Denmark **[25]	Cross sectional survey	Esport athletes	188	17.1 (15 – 35), 97.9% male	Computer	MSK pain during the previous week	Non-standardized questionnaire	Back, neck, shoulder
**Ma (2019), China **[34]	Cross sectional survey	Junior college students	500	17.9 (16—20), 60% male	Smartphone	(1) Positive test for de Quervain’s disease (2) Limited wrist movements in the past 2 weeks	(1) Finkelstein's test (2) Partially standardized questionnaire	Wrist
**Meziat-Filho (2017), Brazil **[35]	Cross sectional survey	High school students	1102	16.8 (14 – 20), 53.3% female	Computer	Neck pain	Partially standardized questionnaire	Neck
**Sekiguchi (2018a), Japan **[42]	Cross sectional survey	School-aged athletes	6143	11^Median^ (6—15), 71.1% male	Computer, console, handheld, smartphone	Current pain in different body parts	Non-standardized questionnaire	Summarized^c^
**Sekiguchi (2018b), Japan **[43]	Cross sectional survey	Young baseball players	200	11 (9—12), 100% male	Computer, console, handheld, smartphone	Elbow or shoulder pain (in throwing arm) during the past 12 months	Non-standardized questionnaire	Elbow, shoulder
**Silva (2016), Brazil **[44]	Cross sectional survey	High school students	961	16.5 (14—19), 61.6% female	Computer, console, handheld	Presence of musculoskeletal pain in the last 6 months	Non-standardized questionnaire	Summarized^c^
**Tazawa (2001), Japan **[36]	Cross sectional survey	School students	1143	NR (6 – 11), 51.2% male	Console	Current or equivocal trapezius stiffness [MS] or MS and displacement of scapula [DS/MS]	Non-standardized questionnaire	Neck, shoulder
**Torsheim (2010), Norway **[40]	Cross sectional survey	School students in Nordic European countries	31,022	11-, 13-, 15-year-olds, 50% female	Computer, console	Physical complaints in past 6 months (backache and headache)	HBSC-Symptom checklist	Back, head
**Wang (2019), China **[26]	Experimental study	College students	12	24.2 (21—28), 58% female	Smartphone	(1) Muscle tension during experiment (2) Musculoskeletal discomfort	(1) Surface electromyography (2) Visual analog	Hand
**Xavier (2015), Brazil **[46]	Cross sectional survey	High school students	954	16.5 (14—19), 61.4% female	Computer, console, handheld	Prevalence of headache	Non-standardized questionnaire	Head
**Yabe (2018), Japan **[45]	Cross sectional survey	School-aged athletes	6441	11^Median^ (6—15), 71% male	Computer, console, handheld	Current pain in different body parts	Non-standardized questionnaire	Back

*CD* Cannot determine, *MSD* Musculoskeletal Disorder, *NR* Not reported, *Yrs* Years

^a^Indicated video gaming devices. Possible devices are computer, console, handheld and smartphone

^b^How MSDs are defined and/or how are changes determined

^c^Results of different body regions were collected independently but presented summarized

^Median^Median was indicated

### Methodological quality

The risk of bias in all studies was assessed by an adapted version of the NOS (Additional file 2) and is shown in Table 2: Risk of bias in individual studies. Ten studies were of high quality with a score of 7 or higher [25, 34, 35, 38–40, 42, 44–46], five studies had a medium quality with a score between 5–6 [26, 33, 36, 41, 43], and one study got score of 1 [37] and was rated as low quality.

**Table 2:** Risk of bias in individual studies

	Selection				Comparability	Outcome		Score
Study: first author (year)	1	2	3	4	5	6	7	
Burke (2002) [41]			★	★	★★	★		5
DiFransiciso-Donoghue (2019) [37]					★			1
Hakala (2006) [38]	★	★	★	★	★★	★	★	8
Hellström (2015) [39]	★	★	★	★	★★	★	★	8
Kang (2003) [33]	★			★	★★	★	★	6
Lindberg (2020) [25]	★	★		★	★★	★	★	7
Ma (2019) [34]	★	★	★	★	★	★	★	7
Meziat-Filho (2017) [35]	★	★		★	★★	★	★	7
Sekiguchi (2018a) [42]	★	★		★	★★	★	★	7
Sekiguchi (2018b) [43]			★	★	★★	★	★	6
Silva (2016) [44]	★	★		★	★★	★	★	7
Tazawa (2001) [36]	★	★			★	★	★	5
Torsheim (2010) [40]	★	★		★★	★★	★	★	8
Wang (2019) [26]				★★		★★	★	5
Xavier (2015) [46]	★	★		★	★★	★	★	7
Yabe (2018) [45]	★	★		★	★★	★	★	7

1: Representativeness of the sample; 2: Justified and satisfactory sample size; 3: Non-respondents rate and characteristics; 4: Ascertainment of the exposure or risk factor (2 stars possible); 5: Comparability of groups based on study design or analysis (2 stars possible); 6: Assessment of the outcome (2 stars possible); 7: Statistical test description; Score: from 0 (low quality) to 10 stars (high quality)

### Results of individual studies

In Table 3: Results of individual studies, the individual results of the included studies are presented. In eleven studies a statistical negative impact was reported of video game playtime [3, 5–7, 9–11, 13, 14, 16, 17] on the musculoskeletal system. In four studies no impact was examined [2, 4, 8, 12] and in one study no effect was found of video game playtime on the musculoskeletal system [44]. Out of the eleven studies, in which a negative impact of video game playtime on the musculoskeletal system was demonstrated, the most reported painful body parts were the neck (n = 4), shoulder (n = 4) and back (n = 3). In ten of these studies odds ratios (OR) were calculated for the dependence of the appearance of musculoskeletal disorders on video game playtime [33–35, 38–40, 42–46]. In order to compare the group of video gamers, in eight studies non-gamers or subjects were used who played less than one hour a day of video games as a control group [33, 38, 39, 42–46]. The remaining studies only contained video gaming time < 2.25 h/day [34] or added every daily hour of video gaming [40] for an OR calculation. In addition, the OR were based on different evaluation methods (Table 1: Study characteristics) and models which are described in the appendix (Additional file 3). A significant increase of the OR was reported in eight studies [33, 34, 38, 39, 42, 43, 45, 46]. In these studies, the OR ranged from 1.3 to 5.2.

**Table 3:** Results of individual studies

**First author (year), country**	**Mean video game playtime (h/week)** **or** **playtime categories**	**MSD affected by playtime?**	**Main findings**	**Comparison group for OR**	**Model 1** **OR (95% CI)**	**Model 2** **OR (95% CI)**	**Model 3** **OR (95% CI)**
**Burke (2002), USA** [41]	3.3 h on Saturday	Not applicable	-Video gaming was a significant predictor for physical complaints (eyestrain, headache, back discomfort, wrist discomfort) -Non-educational games were a significant risk factor for wrist pain, backache and headache -Wrist discomfort was significantly influenced by joystick use and computer gameplay	No OR			
**DiFrancisco-Donoghue (2019), USA** [37]	NR	Not applicable	-The most reported complaints from esport players were eye fatigue (52%), followed by neck, back (41%), wrist (36%) and hand pain (30%)	No OR			
**Hakala (2006), Finland** [38]	≤ 1 h, 2-3 h, 4-5 h, > 5 h/day	Yes	-Risk of NSP increased only in the first model by playing digital games > 5 h/day -Digital gaming exceeding 5 h/day was a threshold for LBP in all three models	Not playing digital games at all or not daily	NSP: 2-3 h: 1.2 (0.9–1.4) 4-5 h: 1.1 (0.7–1.7) **> 5 h: 1.9 (1.2–3.1)** LBP: 2-3 h: 0.9 (0.7–1.2) 4-5 h: 1.1 (0.7–1.9) ** > 5 h: 2.5 (1.5–4.1)**	NSP: 2-3 h: 1.0 (0.8–1.3) 4-5 h: 1.0 (0.6–1.6) > 5 h: 1.5 (0.9–2.6) LBP: 2-3 h: 0.9 (0.6–1.2) 4-5 h: 1.1 (0.7–1.9) **> 5 h: 2.3 (1.3–3.9)**	NSP: 2-3 h: 1.0 (0.8–1.3) 4-5 h: 1.0 (0.6–1.6) > 5 h: 1.4 (0.8–2.4) LBP: 2-3 h: 0.8 (0.6–1.1) 4-5 h: 0.9 (0.5–1.6) **> 5 h: 2.0 (1.1–3.5)**
**Hellström (2015), Sweden** [39]	NR	Yes	-Online multiplayer games were associated with MSK symptoms, but not in multivariate binary logistic regression -Gaming time on weekdays elevated the probability of MSK symptoms significantly	Non gamers vs. online gaming time on weekdays	**1.3 (1.1–1.3)**	Not applicable	Not applicable
**Kang (2003), Korea** [33]	< 1 h, 1-2 h, > 2 h/day	Yes	-MSD prevalence of the upper limbs in gaming room users was 26.8% Most frequently in neck (16.2%), shoulders (14.4%) and wrist (8.8%) -In bivariate analysis a non-significant trend between symptom prevalence of MSDs of the upper limbs and game room usage was observed -In multivariate analysis the duration of game room use was a significant determinant of MSDs in the whole upper limbs and especially in the neck, elbow, wrist and finger areas	Using game rooms < 1 h/day	Neck: 1-2 h: 1.7 (NR) **> 2 h: 2.8 (NR)** Shoulder 1-2 h: 0.8 (NR) > 2 h: 1.2 (NR) Elbow: 1-2 h: 1.1 (NR) > 2 h: 3.5 (NR) Wrist: 1-2 h: 1.5 (NR) **> 2 h: 3.4 (NR)** Finger: 1-2 h: 1.9 (NR) **> 2 h: 4.1 (NR)** Whole upper limbs: 1-2 h: 1.3 (NR) > 2 h: 1.9 (NR)	Neck: **1.8 (1.2–2.8)** Shoulder 1.1 (0.7–1.7) Elbow: **2.2 (1.1–4.7)** Wrist: **2.0 (1.1–3.3)** Finger: **2.3 (1.2–4.6)** Whole upper limbs: **1.5 (1.0–2.1)**	Not applicable
**Lindberg (2020), Denmark** [25]	24.2 ± NR	Not applicable	-MSK pain was prevalent in esports athletes with 42.6% -The most reported body pain regions were the back (31.3%), neck (11.3%) and shoulders (11.3%) -MSK pain was significantly associated with less participation in esports-related training	No OR			
**Ma (2019), China** [34]	< 2 h, 2–3.9 h, 4–5.9 h, > 6 h/day	Yes	-Wrist position and smartphone playtime correlated significantly with DD -Students who spent over 2.25 h/day videogame playing had significantly higher risk of DD -Wrist positioning while smartphone gaming in dorsiflexion was more associated with DD than in function position	Mobile gaming > 2.25 h/day	**3.2 (2.2–4.6)**	Not applicable	Not applicable
**Meziat-Filho (2017), Brazil** [35]	< 2 h, ≥ 2 h/day	Yes	-The prevalence of acute neck pain while playing videogames < 2 h/day was 33.5%, for chronic neck pain it was 16.7% -The prevalence of acute neck pain while playing videogames ≥ 2 h/day was 31.0%, for chronic neck pain it was 13.5%	No OR			
**Sekiguchi (2018a), Japan** [42]	< 1 h, 1-2 h, 2-3 h, ≥ 3 h/day	Yes	-Playing videogames ≥ 3 h/day was significantly associated with MSK pain in crude analysis -The risk of MSK pain was increased by 39% in the group of high videogame time (≥ 3 h/day) in adjusted analysis -The group of high videogame time (≥ 3 h/day) was significantly associated with MSK pain in three or more locations	Video gaming < 1 h/day	1-2 h: 1.0 (0.9–1.2) 2-3 h: 1.1 (0.9–1.3) ≥ 3 h: **1.7 (1.3–2.0)**	1-2 h: 1.0 (0.9–1.2) 2-3 h: 1.1 (0.9–1.3) ≥ 3 h: **1.4 (1.1–1.7)**	Not applicable
**Sekiguchi (2018b), Japan** [43]	< 1 h, 1-2 h, 2-3 h, ≥ 3 h/day	Yes	-High videogame time (≥ 3 h/day) was significantly associated with elbow or shoulder pain in young baseball players -This association was also significant in multivariate analysis	Video gaming < 1 h/day	1-2 h: 1.5 (0.7–3.0) 2-3 h: 1.0 (0.4–2.7) ≥ 3 h: **5.2 (1.6–17.0)**	1-2 h: 1.4 (0.7–3.0) 2-3 h: 1.0 (0.4–2.6) ≥ 3 h: **5.2 (1.5–18.2)**	1-2 h: 1.4 (0.7–3.0) 2-3 h: 0.9 (0.3–2.5) ≥ 3 h: **4.8 (1.4–16.7)**
**Silva (2016), Brazil** [44]	< 1 h, > 1 h/day	No	-The use of electronic games was reported by 2.9% of the adolescents as a triggering factor for at least one pain symptom -The use of electronic games was not associated with pain complaints in any of the surveyed body regions	Electronic gaming < 1 h/day	Cervical region: 1.0 (0.8–1.4) Scapular region: 1.0 (0.8–1.5) Thoracolumbar column: 0.9 (0.7–1.6) Upper limb: 1.1 (0.8–1.5)	Not applicable	Not applicable
**Tazawa (2001), Japan** [36]	0 h, 0.5 h, 1 h/day	Yes	-Excessive console gaming (> 1 h/day) caused greater frequency of MS than non-console playing (25.6% vs. 14.4%) -Console playing correlated highly significantly with MS	No OR			
**Torsheim (2010), Norway** [40]	9.5^a^ ± NR	Yes	-Computer gaming in boys was not correlated with either weekly backaches or headaches -Computer gaming in girls was significantly correlated with weekly backaches and headaches	Per added daily hour of computer gaming	Boys backache: 1.1 (1.0–1.1) Girls backache: 1.1 (1.0–1.1) Boys headache: 1.0 (1.0–1.1) Girls headache: 1.0 (1.0-.1.0)	Boys backache: 1.0 (1.0–1.1) Girls backache: 1.1 (1.0–1.1) Boys headache: 1.0 (1.0–1.1) Girls headache: 1.0 (1.0-.1.0)	Not applicable
**Wang (2019), China** [26]	NR	Not applicable	-Measured median frequency of thumb muscles were reduced significantly after 30 min of continuously playing smartphone games -Measured mean power frequency of thumb muscles were reduced significantly after 30 min of continuously playing smartphone games -VAS scores for discomfort increased significantly after 30 min of playing the smartphone game (score difference: 0 to 2)	No OR			
**Xavier (2015), Brazil** [46]	9.7 ± 15.5	Yes	-Excessive use of electronic devices (> 4 h/day) was associated with the presence of headaches -Excessive use of electronic games (> 4 h/day) was associated with the presence of headaches	Electronic gaming < 1 h/day	1.4 (0.4–5.7)	**1.9 (1.0–3.7)**	Not applicable
**Yabe (2018), Japan** [45]	< 1 h, 1-2 h, 2-3 h, ≥ 3 h/day	Yes	-Videogame-playing time/day was significantly associated with the presence of lower back pain in both crude and adjusted model analyses -In all three models videogame-play times of ≥ 3 h/day were highly significantly associated with lower back pain	Video gaming < 1 h/day	1-2 h: 1.3 (1.0–1.7) 2-3 h: **1.6 (1.1–2.6)** ≥ 3 h: **2.8 (2.0–3.9)**	1-2 h: 1.3 (1.0–1.8) 2-3 h: 1.4 (1.0–2.0) ≥ 3 h: **2.0 (1.4–3.0)**	1-2 h: **1.4 (1.0–1.8)** 2-3 h: **1.5 (1.0–2.1)** ≥ 3 h: **2.2 (1.5–3.2)**

*DD* De Quervain’s Disease, *LSB* Lower Back Pain, *MS* Muscle Stiffness, *MSD* Musculoskeletal Disorder, *MSK* Musculoskeletal, *NR* Not reported, *NSP* Neck-Shoulder Pain, *OR* Odds Ratio; **Bold** = Significant result (p < .05)

^a^The mean value was self-calculated

## Discussion

For the first time, the effect of video game playtime on the musculoskeletal system of video gamers was examined in a systematic review. Sixteen articles met the inclusion criteria of which ten were considered to have a low risk of bias. The main finding of this study is that video gaming could have a negative impact on the musculoskeletal system. With regard to this, video gaming duration was the decisive factor. The odds ratio of musculoskeletal disorders increased up to 5.2 for excessive video game playtime (> 3 h/day).

The first research question can be confirmed, since in eleven out of 16 studies a negative impact of video game playtime on the musculoskeletal system was demonstrated. Seven of these studies were rated with a low risk of bias. The remaining four studies were rated with a moderate risk of bias. Hence, the overall bias of these eleven studies can be rated as low to medium. Consequently, the results confirm that video gaming negatively affects the musculoskeletal system of video gamers.

The second research question, whether excessive video game playtimes can be determined as a decisive factor in the development of MSDs, can also be confirmed. In eight studies a significant increase was reported of OR in individuals with high video game play times. More precisely, the probability of developing a MSD was significantly increased in subjects with video game playtimes of about two to three hours per day or more.

From the results the third research question can also be validated. Video gaming negatively affects different body parts. Out of the eleven studies, in which a negative impact of video gaming on the musculoskeletal system was demonstrated, the most negatively affected body parts caused by video gaming were the neck, shoulder and back. Consequently, video gamers who have excessive playtimes seem to be more vulnerable to developing pain in the neck, shoulder or back area.

### Protective factors against MSDs

According to the results video game playtimes had a negative impact on the musculoskeletal system of video gamers. Moreover, this effect was amplified in individuals who had higher video game play times. However, this effect was not amplified in multivariate models (see Table 3: Results of individual studies). On the contrary, OR tended to decrease in more complex models [33, 38, 39, 42, 43, 45]. Therefore, some factors could have reduced the negative effect of video gaming on the musculoskeletal system. In particular physical activity and age are well-known factors which can influence different health parameters [47–49] and may decrease OR. Physical activity and exercise are factors, which can counteract or prevent MSDs [49]. Moreover, in some studies the health benefits have already been shown of physical activity on the musculoskeletal system in sedentary populations, like video gamers [50]. Additionally, physical activity and even more sport therapy are typical and successful treatments used in the rehabilitation process of MSDs [51]. Consequently, physical activity and exercise could be protective factors which would reduce the negative health impacts of long continuous video game playtimes on the musculoskeletal system. Moreover, these interventions are necessary during the rehabilitation of MSDs if full health and performance status are to be regained of casual and professional video gamers. In addition to these physical factors, also sleeping habits, psychosocial balance, socioeconomic status, and environmental and ergonomic factors can either positively and negatively affect the musculoskeletal system [19, 24, 45]. For this reason, all these factors should be taken into mind, if excessive video game playtimes occur. In addition, video gamers need to be sensitized about the hazards of excessive and continuous video gaming.

### Population and MSDs

The majority of the included populations were school aged kids and young adults in eleven of the 16 studies. In the remaining studies college students (*n* = 3), esports athletes (*n* = 1) and gaming room visitors (*n* = 1) were observed. These populations were domiciled in ten different countries. Hence, cultural, social and psychological differences between the populations might have led to a variation of the reported diseases, pain or risk factors [52, 53]. In addition, some populations are known to be more prone to the development of MSDs. For example, the highest reported OR was 5.2 in crude and adjusted analysis [43]. The authors of this study observed young baseball players, who usually are a vulnerable group for MSDs in the upper limbs [54, 55]. Consequently, just playing baseball can be associated with MSD occurrence. On the other hand, if baseball is the physical activity of choice this could also be a protective factor, which counteracts the negative impact of video gaming. The crude and adjusted OR, which included the number of baseball practice hours among other things, did not differ from each other. Thus, the population group could have had an impact, but it seemed not to be as influential as the video game time in this study. In order to obtain more transparency and a better understanding, studies in which effects of behaviors on the musculoskeletal system are observed, should clearly show the singular impacts in complex statistical analysis. This would provide a better differentiation of the possible influencing factors.

In addition, in the present study the participants age range of the included studies ranged between 6 and 35 years. It is known that higher ages are negatively associated to health related outcomes like MSDs than younger ages [48]. Therefore, it is even more concerning that in the majority of included studies, in which young populations were observed, it was found that there is a negative association of MSDs and video game playtime. In conclusion, high video gaming times can already cause MSDs in children and adolescents. Hence, the early spread of information to parents and kids is necessary about the negative association of MSDs and excessive video game playtimes. On the other hand, specific and tailored prevention and health promotion programs for (young) video gamers are needed.

### Screen-based activities and MSD

As previously mentioned, video gaming is mostly a sedentary activity and comparable with other screen-based activities like office work. Accordingly, different kind of MSDs have been associated to screen-based activities [56–58]. Therefore, several joints could be affected in different ways and with varying severities. The most affected pain regions in this review were the neck (36%), shoulder (36%) back (27%). Due to the heterogeneity in observed gaming devises and the possibility of multiple answers, no specific devise could be connected to one pain region. However, in some studies it has been shown that there is an association of specific screen-based activities with MSDs. It could be demonstrated that watching TV was associated with chronic musculoskeletal pain in Brazilian schoolteachers [59]. The authors showed that an increase in TV watch time by 30 min per day was associated with a 5.1% higher probability of having chronic musculoskeletal pain in the long-term. On the other hand, they also showed that an increase in physical activity by 60 min per week was associated with a 6.2% lower probability of chronic musculoskeletal pain. Therefore, they rated watching TV and physical activity as independent parameters, which effect MSDs in opposite ways.

Smartphone use has been associated with MSDs in the literature. In particular, upper limb [60, 61] and wrist pain [62] are common complaints. Similar results are shown in the present review. In six studies the effect of video game playtime on upper limb joints were analyzed. In four of them a significant increase was reported in OR of disorders or pain in at least one joint caused by excessive video game playing (> 2-3 h/day) [33, 34, 38, 43]. These effects on the upper limbs are not surprising when the biomechanics of smartphone use is visualized, as it requires predominately upper limb activity. Additionally, excessive smartphone use (≥ 5 h/day) has been associated with lower back pain [63]. In contrast, the authors reported that all time intervals of computer use were associated with lower back pain. These results could be explained by the flexibility of smartphone usage compared with computer use. Smartphone use is independent of location and posture, can be executed along with physical activities like walking, and allows, therefore, more mobility and adaptability to delay the beginning of symptoms. In comparison, computer use is mostly a sedentary behavior, which needs a fixed place and thus limits the mobility of users.

Negative associations of neck pain in computer users have also been presented in a systematic review [58]. The relation of continuous computer use and neck pain was significant with constant mouse (> 2 h) or computer use (> 6 h). The authors also reported the associations of stressful and breakless work postures with neck pain. It can be assumed that the combination of physical inactivity, unilateral postures and repetitive movements of the upper limbs are the reasons for this. These results reinforce the findings of our review that video game playtimes of at least two to three hours a day were highly associated with MSDs.

A related analysis of 45,555 pre-adolescents showed the association of screen time in general and the degree of spinal pain [64]. It was found that the relative risk ratio in children increased with increasing hours spent in front of a screen, independent of physical activity. Consequently, screen-based activities could be considered as independent risk factors for MSD. Moreover, plenty of other health risk factors like depression in adults [65] and children [66] or obesity in childhood [67] have been linked to screen-based activities. In summary, not only video gaming, but also other screen-based activities can harm the human musculoskeletal system even if they have different requirements. In particular, continuous computer work and smartphone use were associated with MSDs and other health issues. Consequently, a combination of excessive working and leisure time screen-based activities, could lead to a highly increased risk of developing a MSD in all ages. These problems need to be counteracted at an early age.

### Limitations

The results obtained in this work must be interpreted in the light of some limitations. First, the methodological quality of included studies is heterogeneous. Six out of 16 studies have a moderate to low quality, which must be recognized when interpreting any of the results. In addition, this review just included English articles, which can cause a language bias. Especially articles from countries with a big video gaming community, like China or Korea, could have been beneficial. Second, there is a lack of interventional studies in this research field, which are needed if causality is to be examined. Except for one study, the included studies had a cross-sectional design. In this design a direct causality cannot be assumed. However, if the statistics are adapted using cofactors and covariates, the principles of mediation should allow an association to be made. Third, the generic term musculoskeletal disorders includes different kinds of conditions and diseases. Consequently, there was a high heterogeneity of MSD definitions and an inconsistency of the evaluation methods of the included studies. Due to the versatility of the term MSD there was a lack of standardization of the measurement methods of MSDs. In particular, self-designed non-standardized questionnaires were mainly used for MSD evaluation. Therefore, a generalization of the results is limited because too many different non-validated measurement methods were used.

## Conclusion

In eleven out of 16 studies a negative impact was demonstrated for video game playtime on the musculoskeletal system. Moreover, excessive video game playtimes (> 3 h/day) seemed to be a predictor for the appearance of MSDs. In particular, the neck, shoulder and back were the most negatively affected body regions. The combination of continuous sitting, physical inactivity and repetitive movements of the upper limbs might be the reasons for that. Additionally, other screen-based activities could even amplify this association. Due to the great popularity of video gaming across multiple generations, specific and tailored prevention and health promotion programs for video gamers need to be developed to counteract this immense public health issue. Not only casual video gamers, but also and especially esports athletes should be aware of this if they are to counteract the negative effects of long video game playtimes. For this purpose, it seems reasonable that esports professionals should serve as role models for casual video gamers. In particular, possible correlations between MSDs and different game genres should be observed. Hence, future research should be focused on prevention and health promotion programs for this target group in order to provide a basis for recommendations. Furthermore, studies are needed in which more details of the requirements and burdens are demonstrated on the human body while video gaming.

## Supplementary Information

**Additional file 1.** Search strategy.

**Additional file 2.** Adapted Newcastle-Ottawa Quality Assessment Scale.

**Additional file 3.** OR models of individual studies.

## Data Availability

All the data generated and analyzed during this study are included in this published article and its appendix.

## Abbreviations

GDP: Gross Domestic Product
EMG: Electromyography
MeSH: Medical Subject Headings
MSDs: Musculoskeletal Disorders
NCD: Non-Communicable Diseases
NOS: Newcastle–Ottawa Scale
OR: Odds Ratio
PRISMA: Preferred Reporting Items for Systematic Reviews and Meta-Analyses
RCTs: Randomized Controlled Trials
