# Supplementary material for: Musculoskeletal disorders in video gamers – a systematic review
Source: BMC Musculoskelet Disord. 2022 Jul 16;23:678. doi: 10.1186/s12891-022-05614-0 (PMC9288077; doi:10.1186/s12891-022-05614-0)
Supplement: Supplementary file 1 — Additional file 1. Search strategy. [file 12891_2022_5614_MOESM1_ESM.pdf]

Search strategy

Search number	Term	Keywords in Boolean search format for PubMed	Search mask
1	video gaming	("video gaming"[Title/Abstract] OR "electronic gaming"[Title/Abstract] OR "gamer"[Title/Abstract] OR "gaming"[Title/Abstract] OR "esports"[Title/Abstract] OR "esport"[Title/Abstract] OR "E-sport"[Title/Abstract] OR "E-sports"[Title/Abstract] OR "electronic sports"[Title/Abstract] OR "digital sports"[Title/Abstract] OR "professional gamer"[Title/Abstract] OR "virtual sports"[Title/Abstract] OR "video game*"[Title/Abstract] OR "video gamer"[Title/Abstract] OR "esport athlete"[Title/Abstract] OR "E-sport athlete"[Title/Abstract] OR "esport Player"[Title/Abstract] OR "E-sport player"[Title/Abstract] OR "competitive gaming"[Title/Abstract] OR "console gaming"[Title/Abstract] OR "computer gaming"[Title/Abstract] OR "casual gaming"[Title/Abstract] OR "game streaming"[Title/Abstract] OR "letsplay*"[Title/Abstract] OR "letsplayer"[Title/Abstract] OR "speedrunner"[Title/Abstract] "mobile gaming" [Title/Abstract] OR "handheld gaming" [Title/Abstract] OR ("Video Games"[Mesh]))	Title/ Abstract
2	musculoskeletal	"musculoskeletal" OR "muscle" OR "back" OR "upper back" OR "lower back" OR "neck" OR "shoulder" OR "elbow" OR "wrist" OR "finger" OR "skeletal" OR "osteo" OR "tendon" OR "ligament" OR "connective tissue" OR "fascia*" OR "bone" OR "poor posture" OR "cartilage" OR "lean body mass" OR "body composition" OR ("Musculoskeletal System"[Mesh])	All Fields
3	injuries	"injuries" OR "injury" OR "abuse" OR "damage" OR "trauma" OR "sore" OR "fracture" OR "cramp" OR "strain" OR "epicondylitis" OR "carpal tunnel syndrome" OR "repetitive strain injury" OR "RSI" OR "impingement" OR "herniated disc" OR "slipped disc" OR "arthrosis"	All Fields

4	pain	"pain" OR "backpain" OR "neck pain" OR "pain syndrome" OR "stress" OR "physical stress" OR "discomfort" OR "suffering" OR "agony"	All Fields
5	exclusion	NOT ("elderly" OR "gambling" OR "older adults" OR "office" OR "therapy" OR "treatment" OR "VR" OR "virtual reality" OR "return to play” OR "educat*" OR "mental health" OR "mental disorder*" OR "psychological characteristics" OR "mental stress" OR "addiction" OR "gaming disorder" OR "exergam*" OR "exer-gam*" OR "exer gam*" OR "brain injur*" OR "cognitive disfunction*" OR "ethic*" OR "violen*")	All Fields
6	combined search	#1 AND (#2 OR #3 OR #4 NOT (#5)) AND (2000:2020[pdat])	
