# Supplementary material for: Musculoskeletal disorders in video gamers – a systematic review
Source: BMC Musculoskelet Disord. 2022 Jul 16;23:678. doi: 10.1186/s12891-022-05614-0 (PMC9288077; doi:10.1186/s12891-022-05614-0)
Supplement: Supplementary file 3 — Additional file 3. OR models of individual studies. [file 12891_2022_5614_MOESM3_ESM.docx]

Table 4: OR models of individual studies

| First author (year), country | Statistics and OR calculation | Model 1 | Model 2 | Model 3 |
| --- | --- | --- | --- | --- |
| Hakala (2006), Finland [38] | Simple and multiple logistic regressions:  Weekly NSP and LBP were outcome variables, ICT variables were predictor variables.  Removal limit for variables was 0.1. | All outcomes were adjusted for age and sex. | Outcomes for neck-shoulder pain were adjusted for age, sex, parents’ level of education, school success, timing of puberty, and efficiency of physical activity  Outcomes for low back pain were adjusted for age, sex, school success, and timing of puberty. | Outcomes for neck-shoulder pain were adjusted for age, sex, parents’ level of education, school success, timing of puberty, efficiency of physical activity, and stress symptoms.  Outcomes for low back pain were adjusted for age, sex, school success, timing of puberty, and stress symptoms. |
| Hellström (2015), Sweden [39] | Multivariate binary logistic regression:  Gaming time and motives to play were set in relation to depressive, musculoskeletal, and psychosomatic symptoms. | Outcomes were adjusted for sex, age, body mass index, physical activity, parents’ country of birth, and socio-economic status. | Not applicable | Not applicable |
| Kang (2003), Korea [33] | ANOVA with duncan’s multiple comparison method, chi-square test and chi-square test for trend:  PC game room use was adopted as independent variable. | Crude | Outcomes adjusted for age, duration of game room use per day and perceived subjective stress. | Not applicable |
| Ma (2019), China [34] | Person chi-square test or Fishers exact test:  The strength of correlation between different variables and positive result of Finkelstein’s test was indicated with OR calculation. | Crude | Not applicable | Not applicable |
| Sekiguchi (2018a), Japan [42] | Multiple logistic regression:  To examine the association of game playing time with MSK pain. Additionally multinomial logistic regressions were calculated to evaluate the relationship of game playing time with MSK pain sites. | Crude | Outcomes adjusted for sex (male or female), age (6 to 9, 10 and 11, or 12 to 15years), BMI z-score (continuous variable), years of athletic experience (<1, 1 to <3, or ≥3y), competitive level (low or high), training days per week (≤3 or >3), practice intensity (hard or not hard), studying time (>1, 1, or <1), sleep duration (≤8, >8 to 9, >9), and TV viewing time (≤2, >2 to 3, >3 to 4, or >4) | Not applicable |
| Sekiguchi (2018b), Japan [43] | Multiple logistic regression:  To examine the association of game playing time with elbow or shoulder pain. | Crude | Outcomes adjusted for sex (male or female), age (continuous variable), height (continuous variable), weight (continuous variable), position (pitcher or position player), and number of hours of practice per day on weekdays (≤2 or >2) and weekends (≤6 or >6). | Adjusted for all covariates in Model 2 plus sleeping time (continuous variable) |
| Silva (2016), Brazil [44] | Multiple logistic regression:  The use of electronic games (time) was adopted as independent variable and was associated with the presence of pain. | Sociodemographic variables, physical activity level, nutritional status, and use of electronic devices and their association with complaints of pain in the cervical, scapular, thoracolumbar regions and upper limbs. | Not applicable | Not applicable |
| Torsheim (2010), Norway [40] | Nested model comparison in logistic regression with likelihood ratio test:  Computer games (time) was included in the logistic regression models as a continuous variable. | Outcomes adjusted for country, age, and socio-economic status. | Additively to Model 1, ORs were adjusted for depressed mood, school-related stress, and physical activity. | Not applicable |
| Xavier (2015), Brazil [46] | Multiple logistic regression:  The independent variable electronic gaming time and the presence of a headache were associated. | Comparison of groups with and without headache in association with electronic gaming time. | Factors associated to different kinds of headache: Migraine and high electronic gaming time (>1h/day). | Not applicable |
| Yabe (2018), Japan [45] | Multiple logistic regression:  Video-game-playing time per day was associated with lower back pain. | Crude | Outcomes adjusted for gender, age, BMI, team levels, number of days in practice/week, number of hours practice/day, video-gaming playing time/day, and wake-up time and bedtime. | Outcomes adjusted for gender, age, BMI, team levels, number of days in practice/week, number of hours practice/day, video-gaming playing time/day, and wake-up time and bedtime or sleeping time. |

BMI = Body-Mass-Index; LSB = Lower Back Pain; MSK = Musculoskeletal; NSP = Neck-Shoulder Pain; OR = Odds Ratio.
